# Low-Frequency Sonophoresis: A Promising Strategy for Enhanced Transdermal Delivery

**DOI:** 10.1155/2024/1247450

**Published:** 2024-06-05

**Authors:** Divya Marathe, Vasudeva Sampriya Bhuvanashree, Chetan Hasmukh Mehta, Ashwini T., Usha Yogendra Nayak

**Affiliations:** Department of Pharmaceutics, Manipal College of Pharmaceutical Sciences, Manipal Academy of Higher Education, Manipal, Karnataka 576104, India

## Abstract

Sonophoresis is the most approachable mode of transdermal drug delivery system, wherein low-frequency sonophoresis penetrates the drug molecules into the skin. It is an alternative method for an oral system of drug delivery and hypodermal injections. The cavitation effect is thought to be the main mechanism used in sonophoresis. The cavitation process involves forming a gaseous bubble and its rupture, induced in the coupled medium. Other mechanisms used are thermal effects, convectional effects, and mechanical effects. It mainly applies to transporting hydrophilic drugs, macromolecules, gene delivery, and vaccine delivery. It is also used in carrier-mediated delivery in the form of micelles, liposomes, and dendrimers. Some synergistic effects of sonophoresis, along with some permeation enhancers, such as chemical enhancers, iontophoresis, electroporation, and microneedles, increased the effectiveness of drug penetration. Sonophoresis-mediated ocular drug delivery, nail drug delivery, gene delivery to the brain, sports medicine, and sonothrombolysis are also widely used. In conclusion, while sonophoresis offers promising applications in diverse fields, further research is essential to comprehensively elucidate the biophysical mechanisms governing ultrasound-tissue interactions. Addressing these gaps in understanding will enable the refinement and optimization of sonophoresis-based therapeutic strategies for enhanced clinical efficacy.

## 1. Introduction

Skin is the most significant and approachable part of the body, a protective layer that acts as a barrier to penetration. It comprises 15% of the total body weight and has a surface area of 1.5–2.0 m^2^. It is one of the preferable modes of drug delivery [1–4]. Drug delivery through the skin primarily occurs via diffusion through multiple layers, including the stratum corneum, epidermis, and dermis. Despite its advantages, the efficacy of transdermal drug delivery systems is inherently restricted due to the presence of the stratum corneum. This outermost layer consists of corneocytes, protein-rich flattened dead keratinocytes embedded in a lipid bilayer matrix, serving as a formidable barrier that limits the passage of molecules [2]. However, this limitation can be overcome by different approaches that help transport the drug across the skin. Penetration can be varied or increased using an electrical field, chemicals, and ultrasound. Thus, the transdermal route for delivering drugs is essential and is an alternative method to the oral system of drug delivery (which bypasses the first-pass metabolism) and hypodermal injections (which are painful, have the risk of disease transmission by needle reuse, and also generate harmful medical wastes) [5]. Some of the approaches include microemulsion, transfersomes, invasomes, proniosomes, noisome, nanotransferosomes, supramolecular gel, nanostructured lipid carriers, carbon monotubes, nanocomposites, suction or laser, transdermal patches, topical creams, sonophoresis, iontophoresis, and microneedles [6]. Among the physical methods of drug permeation-enhancing approaches are iontophoresis, microneedles, sonophoresis, and electroporation are more effective [7]. In iontophoresis, the drug substances carrying charge can be moved across the skin using a low voltage current [8]. Microneedles cause temporary disruption, and micron-size pathways will be created across the stratum corneum and epidermis, through which the drug can penetrate easily. Microneedles have some limitations, as they may cause skin irritations and skin allergies. There are also chances of breakage of the needles as the needle size is small and thinner compared to the thickness of the hair follicles on the skin [9, 10]. Electroporation involves high-voltage electric pulses to increase the skin's permeability, causing temporary skin disruption [6]. This method is considered a minimally invasive method [11, 12]. Sonophoresis uses ultrasound for drug permeation through the skin to deliver various drugs, including hydrophilic, lipophilic, and even large molecular-weight compounds [13]. This review discusses the process and multiple applications of low-frequency ultrasound-assisted transdermal delivery.

## 2. Sonophoresis

**Figure 1:**
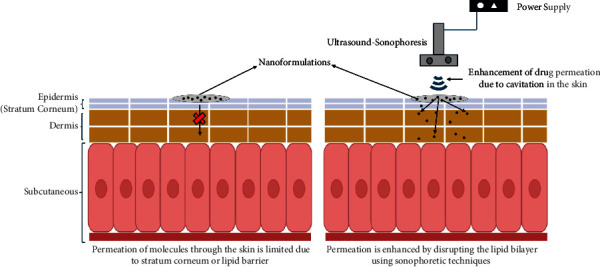
Permeation of drug molecules in the presence of sonophoresis.

Sonophoresis uses ultrasound to deliver the drug molecules across the skin. Ultrasound is the propagation of mechanical energy in a direction parallel to the oscillating particles called longitudinal waves. The ultrasound frequency that increases the skin's permeability ranges from 20 kHz to 16 MHz. Depending upon the frequency used, sonophoresis can be divided into low-frequency (20–200 kHz), intermediate-frequency (0.7–3 MHz), and high-frequency sonophoresis (1–20 MHz). High-frequency and intermediate-frequency ultrasounds are less preferred for drug delivery due to their limited tissue penetration capabilities and higher energy absorption, which leads to potential tissue damage and significant thermal effects. High-frequency ultrasound, with its shorter wavelengths, penetrates tissues shallowly, while intermediate-frequency ultrasound offers slightly better penetration but still struggles to reach desired depths compared to low-frequency ultrasound. Moreover, both frequencies carry the risk of inducing thermal effects and tissue damage, which may compromise the safety and effectiveness of drug delivery. In clinical practice, high-frequency ultrasound is primarily used for diagnostic imaging. In contrast, intermediate-frequency ultrasound finds application in therapeutic modalities such as physical therapy, making their use for drug delivery less practical and potentially hazardous. In contrast, low-frequency ultrasound, ranging from 20 to 200 kHz, stands out for its unique suitability in drug delivery enhancement. Its ability to efficiently penetrate biological tissues with minimal energy attenuation makes it well-suited for enhancing percutaneous drug delivery [14–16]. Additionally, low-frequency ultrasound exhibits favorable industrial and therapeutic applications, such as lithotripsy, liposuction, cataract emulsification, cancer therapy, dental descaling, and ultrasonic scalpels. These attributes render low-frequency sonophoresis particularly advantageous for drug delivery purposes, warranting its specific mention in this context [17]. The exact mechanism involved in sonophoresis is not clear. However, it is thought that cavitation is efficient for improving drug delivery, as cavitation is inversely related to the frequency, whereas low-frequency (<200 kHz) with higher intensity can increase the skin's permeability at a higher rate in response to the pressure changes created due to the movement of the sound waves (Figure 1) [18]. Cavitation includes the formation, growth, and collapse of bubbles, known as inertial cavitation, or slow oscillatory motion of the bubble in an ultrasound field, known as stable cavitation. The bubble's collapse produces a shock wave, affecting the surrounding tissues' structural alteration. Besides cavitation, thermal effects and radiation pressure are other mechanisms in sonophoresis [19]. Sonophoresis equipment comprises several essential components crucial for effective transdermal drug delivery. Typically, it includes an ultrasound generator or transducer responsible for producing ultrasound waves coupled with a handheld applicator connected to the generator. To facilitate the transmission of ultrasound energy from the applicator to the skin, a coupling gel or medium is applied generously to the treatment area postcleansing to ensure optimal contact. The handheld applicator is then maneuvered gently over the treatment area in a circular or linear motion to ensure uniform coverage. Adjustments in intensity, frequency, and duration of ultrasound exposure are made based on treatment requirements and the properties of the target drug or molecule [20]. Throughout the procedure, close monitoring of the skin for any signs of discomfort or adverse reactions is imperative, with adjustments made as necessary to ensure patient comfort and safety. Following the sonophoresis session, excess coupling gel is wiped away, and the skin may be moisturized or treated with postprocedure products as needed. It is crucial to note that proper training and adherence to manufacturer instructions are vital for the safe and effective use of sonophoresis equipment. Additionally, healthcare professionals should consider factors such as patient skin type, medical history, and specific treatment goals when determining the appropriate parameters for sonophoresis therapy. The piezoelectric crystal is the main constituent of sonophoresis equipment, which comprises lead zirconate and converts electrical energy into mechanical energy, generating ultrasonic waves [3]. This noninvasive method has demonstrated enhanced transdermal delivery in both in vitro (morphine, caffeine, and lignocaine) and in vivo (insulin, glucose, mannitol, and heparin) settings, further underscoring its significance in drug delivery innovation [11, 14, 21]. Over several years, many research studies on low-frequency sonophoresis have categorized it into simultaneous and pretreatment sonophoresis. Simultaneous sonophoresis includes the enhancement of transdermal delivery in two ways: the penetration of drug molecules through diffusion by altering the skin structurally and the convectional induction of ultrasound. In the case of pretreatment sonophoresis, ultrasound is applied in the short term, which permeabilizes the skin, and it remains in the condition of high permeability for a longer duration of time, during which the drugs are introduced [1, 22]. Based on the intensity, low-frequency ultrasound can be classified as high intensity or low intensity. Low-intensity treatments are with distorted ultrasonic beams with a spatial and temporal power density of 0.125 to 3 Wcm^2^. High intensity is applied by strongly focusing the ultrasonic beam with a peak power density range of 5 Wcm^2^ to several thousand Wcm^2^ [23, 24]. Low-frequency sonophoresis was used for the local administration of drugs, which has now been used for systemic drug delivery. This method has several benefits: it has high patient compliance to the painless drug delivery, it can also bypass the first-pass metabolism and thereby increase bioavailability, it can control the drug transport rate as it undergoes sustained release of drugs, and it is also possible to deliver the larger molecules, which is a limitation of subcutaneous injection [25]. In vivo, many therapeutic macromolecules, such as insulin and low-molecular-weight heparin [23] [26], and vaccines, have been delivered using low-frequency sonophoresis. It has also been key in administering peptides and proteins [27]. Low-frequency sonophoresis has also been used for cosmetic purposes, skin rejuvenation, and cellulite remediation. Low-frequency sonophoresis was first used clinically, involving the delivery of liposomal lidocaine, which decreased the duration of onset of action for local anesthesia. Other studies have shown that the delivery of histamines [25], 5-fluorouracil, ascorbic acid [26], epinephrine, and cyclosporine solution also utilized low-frequency sonophoresis [28, 29]. A study performed by Meissner and his team compared the effect or influence of ultrasound parameters (low or high frequency) on the release of ibuprofen from hydrogel (alginate and poloxamer) matrix, which proved that an increase in drug release behavior from the gel matrix at low frequency as compared to high-frequency ultrasound [30]. Another study demonstrated groundwork by utilizing sonophoresis in delivering biomacromolecules from polymeric nanocarriers via transdermal delivery. This study introduces a novel transdermal delivery system using sonophoresis coupled with carboxymethyl chitosan nanocarriers for administering basic fibroblast growth factor (bFGF), overcoming its inherent instability and enabling widespread application on damaged skin. Experimental findings demonstrate enhanced bFGF penetration facilitated by low-frequency ultrasound, underscoring the potential of polymeric nanocarriers and sonophoresis for efficient delivery of biomacromolecules in skincare therapeutics (Table 1) [40]. The exploration of low-frequency sonography (LFS) as a potent method for enhancing skin permeability surpassed traditional ultrasound frequencies, prompting a quest to dissect the mechanisms underlying LFS-induced skin perturbation. Notably, these mechanisms vary depending on the treatment approach adopted. Two primary approaches are the simultaneous method, where the drug is incorporated into the LFS coupling medium, and the pretreatment method, involving LFS application followed by passive drug delivery through a patch or formulation. Recent studies favor the pretreatment method due to limitations of the simultaneous approach, such as drug compound degradation. However, advancements in miniaturized LFS devices with degassed coupling mediums have revived interest in the concurrent approach [49].

Early investigations employing acoustic spectroscopy pinpointed inertial acoustic cavitation as the pivotal mechanism for enhancing skin permeability via LFS. Studies by Tang et al. and Tezel et al. confirmed that cavitation above the skin, particularly in the aqueous coupling medium, drives the enhancement process. Furthermore, the research identified transient cavitation microjets directed at the skin surface as the likely contributor to enhanced permeability. While cavitation within the skin can impact therapeutic ultrasound frequencies, it does not significantly strengthen skin permeability with LFS. Nevertheless, confusion persists in distinguishing these mechanisms, leading to erroneous citations in recent literature. In addition to cavitation, the simultaneous protocol introduces convective processes as potential contributors to enhanced drug transport, particularly evident in heat-stripped skin models. However, the role of convection in LFS-mediated transdermal delivery remains uncertain, with conflicting findings across numerous studies [50–53].

A paradigm shift occurred in understanding LFS mechanisms when researchers observed that skin permeability enhancement predominantly occurs in localized regions rather than uniformly across the skin surface. These highly perturbed areas, termed localized transport regions (LTRs), exhibit significantly higher permeability than untreated skin. Even less perturbed regions, referred to as non-LTRs, demonstrate enhanced transport properties when introducing a chemical enhancer like sodium lauryl sulfate (SLS) in the LFS coupling medium. Recent investigations highlight distinct enhancement mechanisms within LTRs and non-LTRs. LTRs show strong frequency dependence in pore radii, suggesting a frequency-dependent process like transient cavitation microjets' collapse drives enhancement within LTRs. At the same time, SLS's frequency-independent action primarily enhances non-LTRs [15, 16, 54, 55].

Understanding the impact of physiological variations in skin and mucosa on sonophoresis application is crucial for elucidating its efficacy and safety across different contexts. These variations encompass factors, such as the skin's location, its integrity (whether intact or injured), and the application of sonophoresis to mucosal surfaces. Here, we delve into each aspect to comprehensively understand their significance.

### 2.1. Location-Specific Effects

The physiological characteristics of skin vary depending on its location on the body. For instance, the skin on the face differs from that on the palms or the back in terms of thickness, sebum production, and vascularity. Such variations can influence the response to sonophoresis, affecting factors like drug penetration and tissue response. Therefore, investigating how sonophoresis behaves across different anatomical sites is imperative for tailoring its application to specific therapeutic goals.

### 2.2. The Integrity of the Skin Barrier

The integrity of the skin barrier plays a pivotal role in determining the efficacy and safety of sonophoresis. Intact skin poses a formidable barrier to drug penetration, whereas compromised skin, such as wounds or lesions, may exhibit altered permeability. Sonophoresis applied to injured skin must be approached cautiously, considering factors like inflammation, tissue repair processes, and susceptibility to adverse effects. Moreover, understanding how sonophoresis influences wound healing is essential for clinical utilization in wound management.

### 2.3. Mucosal Applications

Extending beyond cutaneous delivery, sonophoresis holds promise for enhancing drug delivery across mucosal surfaces, such as the oral cavity, nasal passages, and vaginal epithelium. However, mucosal tissues present distinct physiological characteristics compared to skin, including higher permeability and susceptibility to irritation. Addressing the unique challenges and opportunities associated with mucosal sonophoresis is essential for expanding its therapeutic repertoire and ensuring patient safety.

By systematically investigating the interplay between sonophoresis and physiological variations in skin and mucosa, we can optimize its application for diverse therapeutic objectives while minimizing risks associated with treatment. Moreover, elucidating the underlying mechanisms governing these interactions will pave the way for developing targeted strategies tailored to specific anatomical sites and clinical indications [16, 49, 51, 52, 54, 56].

## 3. Mechanism of Sonophoresis-Mediated Delivery

Many investigations on sonophoresis have shown that the mechanism involved is unclear. However, several phenomena occur when exposed to ultrasound. These include cavitation effects, thermal effects, convective transport, and mechanical effects.

### 3.1. Cavitation Effects

**Figure 2:**
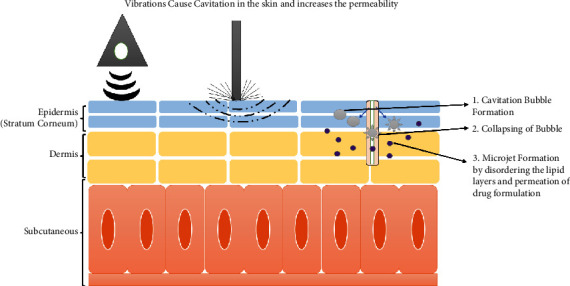
Disruption of stratum corneum to increase permeability by using ultrasound.

The mechanism of low-frequency sonophoresis usually deals with cavitation. It is the process of the formation of gaseous cavities like bubbles and their collapse. Usually, in low-frequency sonophoresis, the cavitation is induced in a coupled medium, the liquid between the ultrasound transducer and the skin (Figure 2). Coupling medium can also serve as a vehicle for the drug. The coupling medium agents include mineral oil, water-miscible creams, and gels. Cavitation inside the stratum corneum occurs in keratinocytes, lipid bilayer, or both. Generally, cavitation occurs in keratinocytes, which contain high amounts of water, as in this process, the ultrasound deals with the dissolved air content in the surrounding buffer. The maximum radius formed by the cavitation bubble depends on the frequency and the pressure amplitude. For low-frequency sonophoresis, the frequency is 20–100 kHz, and the pressure amplitude ranges from 1 to 2.4 bars. The maximum radius of the bubble is 10–100 *µ*m. There are two types of cavitation: stable cavitation and inertial cavitation. Stable cavitation is related to the periodic growth and oscillation of the bubble, whereas inertial cavitation is associated with the violent growth and collapse of the cavitation bubble [57, 58].

Overall, inertial cavitation depends on the intensity of the ultrasound. The ultrasound intensity above the threshold is needed before establishing inertial cavitation. This threshold value is related to the pressure amplitude, which is necessary for the growth and collapse of cavitation nuclei. Beyond this threshold, the activity of inertial cavitation rapidly decreases in terms of ultrasound frequency. This relation states that inertial cavitation becomes difficult with increasing ultrasound frequency. These data indicated that inertial cavitation plays a significant role in low-frequency sonophoresis. Inertial cavitation enhances the permeability of the stratum corneum by the symmetrical collapse of the bubble, causing the emission of shock waves (amplitude of shock wave decreases with an increase in distance), which leads to the disruption of lipid bilayer in the stratum corneum. The effects of the ultrasound field cause the migration of cavitation bubbles toward the boundary and collapse. This collapse occurs asymmetrically, leading to the formation of microjets that project toward the surface. The disruption of the lipid bilayer increases the solute diffusion coefficient. At higher levels, the disruption may cause the permeation of the coupling medium into the stratum corneum [59, 60]. Therefore, several investigations have been reported where the coupling medium comprised the aqueous solutions of surfactants, which leads to the penetration of water and surfactants into the lipid bilayer of disrupted stratum corneum; this helps the further promotion of disruption and opening of the pathway for the permeation of solute [14, 23]. Another study investigated how skin texture density affects individual cavitation bubbles' behavior under low-frequency ultrasound, which is crucial for improving transdermal fluid extraction and drug delivery. Researchers used high-speed photography to study bubble dynamics near skin-like surfaces with varying textures. They found that texture density significantly influences bubble size, movement toward the surface, collapse time, and jet speed upon collapse, particularly with ultra-high texture density. These findings shed light on how skin texture structure enhances acoustic cavitation, offering insights for related applications [61]. Concurrently, the development of a conformable ultrasound patch aims to boost transdermal absorption of niacinamide, demonstrating a substantial 26.2-fold increase in drug transport. This innovative device holds promise for effective and safe large-area applications in skin care and cosmetic therapy, presenting a significant advancement in transdermal drug delivery technology [62].

### 3.2. Thermal Effects

Thermal effects induced by sonophoresis play a significant role in determining its safety and efficacy. When ultrasound waves are applied to tissues, they are absorbed, leading to an increase in the temperature of the surrounding medium. This temperature elevation is particularly relevant in scenarios where tissues have a high protein content, necessitating higher ultrasound intensities, or in areas with poor vascularization, as the heat dissipation is limited. Additionally, when bones are involved in the heated volume, they experience more significant thermal effects than muscle tissues due to their higher ultrasound absorption coefficient and density. The extent of temperature increase in the medium is directly proportional to both the intensity of the ultrasound and the duration of exposure. Furthermore, as the temperature of the medium rises in terms of ultrasound frequency, the absorption coefficient of the medium also increases. This relationship underscores the importance of monitoring and controlling the temperature during sonophoresis procedures to prevent adverse effects on the skin and underlying tissues. A critical parameter to consider in managing thermal effects is “time to threshold.” This refers to the duration after the threshold temperature is surpassed and becomes crucial for ensuring the safety of tissue exposure to ultrasound. By understanding and adhering to safe thresholds, healthcare professionals can mitigate the risk of thermal damage and optimize the therapeutic benefits of sonophoresis [16, 23, 54, 63].

### 3.3. Convective Transport

The hindrance of the incident and reflected ultrasound waves in the diffusion cell and the oscillation of the cavitation bubble cause the generation of fluid velocity in the porous medium, which affects the transdermal transport, inducing convective transport through the skin, hair follicles, and sweat ducts. Some investigational evidence shows that convective transport does not show much influence on the transdermal permeation of drugs [64, 65].

### 3.4. Mechanical Effects

Ultrasound is a longitudinal pressure wave that induces sinusoidal pressure variation in the skin, which then induces sinusoidal density variation. The density variation occurs rapidly when the frequency is above 1 MHz, which affects the growth of the nucleus (no growth) and ceases cavitation. The generation of cyclic stress is another effect of density variation, which leads to the fatigue of the medium. These stresses disrupt the lipid bilayer very quickly, which increases permeability. However, this increase in permeability is unessential; thus, mechanical effects are not significant in the therapeutic aspects of sonophoresis [63, 64]. Therefore, the cavitation method is the most considerable mechanism in developing permeation of the barrier, stratum corneum, in the sonophoresis.

## 4. Pharmaceutical and Biomedical Applications of Sonophoresis

Sonophoresis-assisted transdermal delivery is used in various applications, as discussed in the following sections. Table 1 gives a list of some of the applications.

### 4.1. Transport of Hydrophilic Drugs

In discussing the various approaches for enhancing the transport of hydrophilic drugs, it is essential to consider studies that have compared different methods to elucidate their comparative efficacy. While several strategies exist, such as chemical modifications of the drug, saturation techniques, and iontophoresis, it is imperative to examine how these methods stack up against each other to enhance skin permeability and drug delivery efficiency. Chemical and physical enhancers offer promising avenues for decreasing skin barrier properties, with surfactants and oils acting as chemical enhancers by denaturing keratinocytes or disrupting lipid bilayers, and physical enhancers like iontophoresis, electroporation, thermal ablation, liquid jet injectors, and sonophoresis providing alternative mechanisms for enhancing drug penetration. Notably, low-frequency sonophoresis has emerged as particularly advantageous among these methods. Studies have demonstrated that low-frequency sonophoresis at 20 kHz can induce penetration of hydrophilic drugs up to 3000-fold greater than other techniques, highlighting its potential as a superior approach for transdermal drug delivery. Furthermore, exploration and comparison of these methods in clinical and experimental settings can provide valuable insights into optimizing drug delivery strategies for enhanced therapeutic outcomes [66]. The main mechanism of sonophoresis, cavitation, leads to permeation enhancement, allowing hydrophilic drugs to transport. In low-frequency sonophoresis, the extent of enhancement can be controlled by altering the time and other parameters of the ultrasound. The size of the pores created on the skin using low-frequency sonophoresis can also be controlled by adjusting the intensity and frequency of ultrasound. Moreover, synergistic effects can also be used between physical and chemical enhancers (low-frequency sonophoresis and surfactants) to increase the permeability of the drug molecules. As the ability to enhance the skin permeability of the chemical enhancers is weak, a second enhancer and physical enhancers can be used to enhance the skin permeability and improve the penetration of the first enhancer. Therefore, it has been said that the synergistic effects between low-frequency sonophoresis and surfactants are connected with the clinical viability of sonophoresis. However, this synergistic effect is not entirely understood as no physical mechanisms are proposed on how low-frequency sonophoresis increases the permeation of surfactants [49, 53].

The aqueous porous pathway model was introduced to describe the transdermal transport of hydrophilic molecules through low-frequency treated skin. The hydrophilic molecules will be assumed to permeate through the aqueous pathway that passes over the stratum corneum. The aqueous porous pathway model has a relation between the permeability of the skin to hydrophilic molecules, P, and skin electrical resistivity, R. Using this relation for the diffusion of hydrophilic molecules through the aqueous porous pathway or pore gives a linear decline of log*P* vs log*R* with slope −1. By substituting the known values of parameters, one can calculate the essential structural parameters of the skin using this aqueous porous pathway model [13]. Large pore size is formed in the localized transport regions due to cavitation effects, and this localized transport area is inversely related to the skin resistivity R. Therefore, the samples treated with higher low-frequency sonophoresis intensity have lower log*R* values. The aqueous porous pathway model plays a vital role in finding information on how different low-frequency sonophoresis treatments are authorized, in both the presence and the absence of chemical enhancers, which affect the structural conditions of the skin. Therefore, the aqueous porous pathway model helps us to understand and describe the transport of hydrophilic molecules across the skin, and it also helps to understand the structural conditions of the skin on low-frequency sonophoresis treatment [31, 49, 67, 68].

### 4.2. Transport of Macromolecules

The use of low-frequency sonophoresis to transport high-molecular-weight proteins has benefits as it bypasses the harsh environment of the gastrointestinal tract, which decreases the therapeutic activity and protein denaturation. Therefore, low-frequency sonophoresis (20 kHz, 225 mW/cm^2^, 100 ms pulse every second) is a suitable method for transporting the high-molecular proteins as this method allows the controlled release of the molecules [63]. The first tested protein molecules are insulin and IFN-*γ*. High-molecular-weight proteins can be transported through low-frequency sonophoresis and high-molecular-mass drugs, fibers, hormones, biopolymers, liposomes, nanoparticles, oligonucleotides, and vaccines. The protein most studied in transport due to low-frequency sonophoresis is insulin because of its high importance in treating diabetes. According to the studies, low-frequency sonophoresis-mediated insulin delivery effectively lowered the blood glucose levels in diabetic hairless rats (400 to 200 mg/dl in 30 min), above the threshold ultrasound intensity and treatment time. Many studies have been reported on using pretreated-type sonophoresis to transport macromolecules. The degree of skin permeabilization of this method should be determined before the drug delivery. This was done by using ultrasound on the skin and measuring the conductance. Pretreatment of the skin by low-frequency ultrasound enhanced the release of insulin [22, 49]. A study performed by Park and the team developed ultrasound-responsive liquid core nuclei, which helped deliver niacinamide and adenosine through transdermal drug delivery and showed an 11.9- and 7.33-fold increase in permeability, respectively. Thus, this technique can be used widely for different skin applications [69]. Zhu et al. used a new combined system of low-frequency sonophoresis with an ultrasonic transducer and motor for calcein delivery, showing that a change of ultrasonic motor with static pressure can effectively control noninvasive permeability by adjusting motor driving amplitude voltage and duty ratio [70]. A study performed by Zhai and team used low-frequency sonophoresis for transdermal delivery of heparin with sponge spicules for the treatment of venous thrombosis, which confirmed that utilization of this combined technique significantly increases the transdermal delivery and is helpful for transdermal delivery of other hydrophilic macromolecules [71].

### 4.3. Gene Delivery

Gene delivery through physical methods requires transferring the therapeutic materials into the cell. One such physical method is sonophoresis. Ultrasound was used to enhance the gene transfer into the cell and was suggested to be a potential physical method [72]. The most widely used application of sonophoresis as a topical enhancer is in the field of gene therapy. Gene therapy is a technique used to correct the defective genes responsible for the development of diseases or to replace the abnormal disease-causing gene with the normal gene. The delivery of genes for skin or muscles can be done by electroporation [66] and the biolistic (gene gun) method [73]. However, it is problematic for delivering genes to deep organs like the liver, where an incision is required to place electrodes in electroporation. Compared to electroporation, sonophoresis, as an ultrasound, can be applied to the skin and focused intensely on the tissues. Disruption of the cell membrane and vessel by exposure to ultrasound and the formation of microbubbles increase the permeability and delivery of the gene, allowing the target cells to produce their therapeutic proteins [74]. Ultrasound and microbubbles play a significant role in aiding transfection, a process of introducing nucleic acid into eukaryotic cells by nonviral method. Ultrasonic waves are used for in vivo studies on the transport of naked DNA into muscles, the liver, the kidney, the heart, the carotid artery, solid tumors, and transdermal delivery. Experiments were conducted by co-administering the microbubbles and plasmid DNA with the genes coding marker proteins [10, 41]. This experiment showed that applying ultrasound, microbubbles, and plasmid improved transfection efficiency compared to naked plasmid DNA alone. A carrier molecule, a vector, delivers the gene to the target cell. Cutaneous gene therapy is used for the most common disease, severe genodermatoses, including epidermolysis bullosa and ichthyosis. Other applications include healing cutaneous wounds, such as severe burns and skin wounds of diabetic patients [14]. Gene therapy permeates larger molecules, such as macromolecules with many base pairs weighing one million Daltons through the skin. Skin permeability can be increased by pretreating the skin with ultrasound and then allowing the delivery of the carrier molecule or vector-gene complex [75]. Gene delivery by viral vectors is efficient but causes problems such as immunogenicity of viral proteins, potential mutagenicity due to integration of viral sequence, and low efficiency for systemic delivery. However, nonviral or naked plasmid (pDNA) is much safer and has several advantages. Ultrasound is also used for improving the transport of DNA trapped in nanoparticles, liposomes, and micelles [76]. There is a new method called electro-sonoporation, which combines sonophoresis with electroporation. This consists of an intramuscular injection of naked DNA, followed by 5 minutes of ultrasound administration, combining the electrical pulse. This method showed greater efficiency when the electrical pulse was added between ultrasonic wave deliveries [25, 77–79].

### 4.4. Vaccine Delivery

Several drug delivery techniques are used for vaccine delivery; sonophoresis is also one of the methods to deliver the vaccines across the skin. Transdermal vaccination has several advantages over hypodermic needle-based transfer, which include ease of administration, no risk of disease transmission, and patient compliance to painless drug delivery. The first low-frequency sonophoresis vaccine delivery study was reported to deliver immunization for tetanus toxoid into an in vivo mouse model. Examinations were conducted on the effect of the ultrasound cycle (ultrasound wave measuring 20 kHz for a total period of 45 sec) and sodium dodecyl sulfate and its concentration on immune responses. The applied ultrasound had a duty cycle of 10% or 20%, 0.1 sec on and 0.9 sec off, and 0.2 sec on and 0.8 sec off. On exposure to ultrasound, mice were immunized with tetanus toxoid consisting of thirty flocculation units. It was demonstrated that IgG titers remained undetectable in the absence of ultrasound. However, pretreating the mouse's skin with ultrasound increased the levels of antitetanus toxoid IgG and protective toxins neutralizing antibodies. However, the responses were less than 30-fold compared to the intramuscular immunization. It was also hypothesized that enhanced vaccine delivery is possible through two prolonged mechanisms: activation of immune cells following sonophoretic transcutaneous immunization and facilitated transcutaneous vaccine delivery. The nano-sized cavitation nuclei promote inertial cavitation at moderate pressure amplitudes and frequencies, which has been found to help increase the application. Transcutaneous immunization is a potent novel technique as topical immunization provokes systemic and mucosal immunity. Two mechanisms explain why skin pretreatment with low-frequency ultrasound before contact with antigen vaccine enhances the immune response. First, there is an increase in the vaccine delivery on ultrasound pretreatment, thus enabling the skin's immune response. The second mechanism involves Langerhans cells (Langerhans cells are essential for T cell-mediated immune response) and immune cells of the skin, which trap the antigen and present it to the immune system. The mechanism of immune response by low-frequency sonophoresis is unclear [78, 80]. Hu and team developed a monitorable and programmable intradermal delivery for vaccine using an ultrasound perforation array, which confirmed the successful perforation with higher permeation and skin expression. Thus, it holds tremendous potential for intradermal vaccination [81].

### 4.5. Carrier-Mediated Delivery—Nanocarriers

Nanocarriers are smaller-sized particles with the advantage of having a longer time of circulation and higher diffusivity through the tissue. Due to their smaller size, they can be carried by a cell and pass through the blood capillaries to deliver the drug [82]. Nanocarriers should be small enough to pass through the blood capillaries and large enough to withstand renal excretion, and they should also be stable to prevent biodegradation until they get activated by ultrasound [83]. Nowadays, ultrasound contrast for ultrasound imaging is used as a drug carrier, such as nanobubble. The drug delivery will be feasible using ultrasound as it is applied only for a particular region, less time, and involves controlled release. Drugs can be loaded into the bubble in two ways: by associating the drug with the superficial layer of the bubble and by encapsulating the shell into the core of the bubble containing the oil reservoir. Drugs can also be packed with nanoparticles, which can get attached to the surface of the microbubbles. There are four types of bubbles used for ultrasound-mediated drug delivery with nanocarriers. Those are drug-loaded delivery on exposure to ultrasound, in situ formed nanodroplets, acoustically active nanobubbles, and targeted bubbles [84]. According to some studies, nanocarriers include micelles, liposomes, nanoemulsion, and polymer-based nanoparticles. Many inorganic nanocarriers, such as metal nanoparticles, silica-based nanovehicles, and carbon-based nanovehicles, are used. Some nanocarriers studied are liposomes, micelles, dendrimers, and nanoemulsions.

#### 4.5.1. Micelles

Amphiphilic molecules are associated with a hydrophobic core surrounded by a hydrophilic ring, which forms micelles on exposure to the aqueous phase. This association is mediated by hydrophobic forces and by electrostatic force of attraction. Micelles are 10–100 nm in diameter and can store hydrophobic drugs in its core. Usually, the hydrophobic core is a polymer, and Pluronic® is the most common copolymer used in ultrasound-mediated drug delivery [34]. Above critical micellar concentration, the hydrophobic drug gets partitioned in the nonpolar medium of the micelle body (hydrophobic core) in the aqueous solution, and the drug is released when the solution is diluted below the critical micellar concentration. Several studies have shown that the release of drug load when exposed to ultrasound is correlated with the induction of ultrasound cavitation [85]. Experiments demonstrate that the use of low-frequency sonophoresis (20 kHz) increased the efficiency of drug release of the anticancer drug doxorubicin, and the efficiency of drug release decreased by increasing the frequency [37]. It is also demonstrated that the application of ultrasound causes the release of doxorubicin from the micelles [44].

#### 4.5.2. Liposomes

Liposomes are the carriers used for in vivo drug delivery with 10–100 nm diameter containing an aqueous core surrounded by a lipid bilayer larger than micelles. The phospholipid-bilayer mimics the cell membrane and helps in loading the lipophilic drugs. Similarly, the hydrophilic drugs are loaded into the aqueous core [85, 86]. Liposomes are used to deliver insoluble anticancer drugs like doxorubicin [87]. Several studies demonstrate that ultrasound enhances the drug's release from liposomes. Cavitation, thermal effects, and acoustic streaming are the mechanisms involved in the drug release from the liposomes involving ultrasound. Generation and sudden collapse of the cavities or bubbles formed near the lipid bilayer of the liposomes trigger the release of the drug. Some experiments showed that low-frequency ultrasound enhances the release of the internal drug load of the liposomes [88]. Studies state that cavitation is vital in releasing doxorubicin from the liposomes. To prevent the changes in the temperature due to exposure to ultrasound, a high-frequency ultrasound of 1 MHz is used to introduce the doxorubicin-carrying liposomes [89]. A study showed that effective drug delivery can be achieved when liposomes are coupled with ultrasound, as sonication breaks down the lamellae. Here, the in vitro drug release profile and in vivo systemic availability of anti-inflammatory activity of diclofenac in liposome as an ointment base was administered by the application of ultrasound, which showed that there was slow systemic availability in the site of application and pulsed drug systemic levels were achieved on ultrasound application [46].

#### 4.5.3. Dendrimers

Dendrimers are monodispersed, repeatedly branched, three-dimensional macromolecules and are the effective permeations and solubility enhancers. Experiments have shown that dendrimer-coupled sonophoresis has increased the permeation of analgesic drug diclofenac by 16.5-fold. Low-frequency ultrasound was applied before gel was applied to the skin, and the main aim was to determine the effect of ultrasound used in the permeation of diclofenac. It was shown that the permeation of diclofenac on the skin increased by increasing the ultrasound application time [4, 47].

### 4.6. Synergistic Effect of Sonophoresis with Other Techniques/Permeation Enhancers

#### 4.6.1. Sonophoresis with Iontophoresis

Iontophoresis is one of the transdermal drug delivery techniques that involve the transport of charged drug substances across the skin using low voltage current. This method is commonly studied in combination with low-frequency sonophoresis [90]. The experiment used heparin, an antithrombotic drug, as a model molecule. Ultrasound was applied on the skin before the application of iontophoresis and showed the enhancement of penetration 56-fold higher than iontophoresis alone (3-fold) and sonophoresis alone (15-fold). The experiment was conducted between positively and negatively charged surfactant sodium lauryl sulfate and dodecylpyridinium chloride. In another experiment, it was shown that a combination of iontophoresis with low-frequency sonophoresis was used to increase the in vitro permeation of sodium nonivamide acetate, where the use of sonophoresis decreases the current required to achieve the drug flux in the presence of an electric field [91]. One of the studies showed that when combining sonophoresis and iontophoresis, the penetration of the water-soluble antipyrine sodium salicylate increased ten times more than that of iontophoresis and sonophoresis when applied alone [92].

#### 4.6.2. Sonophoresis with Chemical Enhancers

Combining sonophoresis and chemical enhancers like surfactants increases transdermal drug transport. Chemical enhancers dissolve the skin's lipid layer by denaturing the corneocytes, which increases the skin's permeability. However, the effect increases when using sonophoresis with these chemical enhancers. Studies have shown that sodium lauryl sulfate combined with sonophoresis increased mannitol's transdermal transport by 200-fold. When used alone, sodium lauryl sulfate increased 3-fold, and sonophoresis alone showed an enhancement of 8-fold [93]. Another study has demonstrated the delivery of hydrocortisone by using high-frequency sonophoresis and chemical enhancer laurocapram. The permeation into the skin was due to the diffusion of laurocapram by the thermal effects resulting from the application of ultrasound [94].

#### 4.6.3. Sonophoresis with Microneedles

Sonophoretic enhanced microneedles array (SEMA) is a method that combines ultrasound and microneedle type of permeation enhancers, which has an improved effect on transdermal drug delivery. In the SEMA method, the microneedle penetrates the stratum corneum without pain and bleeding and then delivers the drug to the epidermis. In contrast, the low-frequency sonophoresis enhances transdermal drug transport by causing cavitation in the epidermis of the skin. SEMA devices contain two parts: an ultrasonic emitter and a microneedle array. A study proved that passive diffusion of hydrophilic calcein is not possible. However, increasing the skin's permeability by SEMA showed an effect of 9-fold compared to the enhancement by microneedles alone, which showed effects of 5-fold, and ultrasound alone showed an impact of 8-fold [95]. One of the studies was conducted to determine the combined effect of lidocaine and polymer-based hydrogel formulation with the help of microneedles and ultrasound. It showed that using the combined effect of microneedle and ultrasound pretreatment increased lidocaine's permeability [96].

#### 4.6.4. Sonophoresis with Electroporation

In electroporation, the skin structure is altered by applying the electric field, creating an aqueous pathway in the lipid bilayer. Convective transport in an electric field and ultrasound enhances transdermal transport. A study has shown with the use of ultrasound in the model of calcein and sulforhodamine, the threshold voltage reduces, which is required for the transport of calcein and sulforhodamine in the presence of an electric field [97].

### 4.7. Ocular Drug Delivery Using Sonophoresis

The eye cornea is considered the major pathway for the permeation of drug substances, which is applied topically. It comprises three avascular primary layers: epithelium, stroma, and endothelium. Epithelium is the prevailing barrier for transporting hydrophilic drugs, and stroma prevents the entry of lipophilic drugs. Physical enhancers like iontophoresis and sonophoresis help overcome the cornea's barrier properties. In sonophoresis, the corneal permeability of *β*-blockers can be increased by applying the ultrasound for 5 minutes, having a frequency of 470–880 kHz and intensities ranging from 0.2 to 0.3 W/cm^2^ [98]. The mechanism involved was thought to be the cavitation-induced corneal damage, which healed in 6 hours [99, 100]. Studies have shown that ultrasound increases the permeability in glaucoma patients, where drugs of different lipophilicities are delivered [100].

### 4.8. Sonophoresis-Mediated Nail Drug Delivery

Sonophoresis is used as a nail drug delivery system by increasing the permeability of the nail in the treatment of onychomycosis, a nail fungal disorder where the nail thickens and appears yellow, and psoriasis. Usually, oral antifungal drugs are used to treat onychomycosis, and corticosteroid injections are given into the nail folds for treating psoriasis. However, the efficacy of the topical delivery is reduced due to the poor permeability of the nail plate. Only a few nail enhancers are identified: N-acetyl cysteine, N-(2-mercaptopropionyl), mercaptoethanol, and glycine [101]. Onychomycosis is of particular concern for patients who have diabetes, as those patients have risks of developing ulcers, gangrene, and cellulitis. The most common oral drug for the treatment of onychomycosis is terbinafine. However, this drug takes more than six months to show its action, and it has a 30% failure rate with many side effects [102]. Another drug used is ciclopirox, which is applied in the dorm of nail polish and has a cure rate of 36% after applying for six months daily. The drug binds to the keratin, which decreases the drug's ability to permeate through the nail or decreases the amount of drug transferred. Applying ultrasound of 800 kHz and 1 MHz to the nail increases the amount of drug permeation, thus allowing the drug to reach the treatment site. Streaming and cavitation enhance drug delivery using ultrasound through the nail [103, 104].

### 4.9. Drug and Gene Delivery to the Brain

Treatment of neurodegenerative diseases is very challenging due to the presence of the blood-brain barrier, which limits the permeation of the drug into the brain. The blood-brain barrier can be briefly disrupted using low-intensity focused ultrasound and microbubble formation [105]. Alzheimer's disease is one such neurodegenerative disease that is caused by the accumulation of proteins (amyloid) in the brain cells, forming plaque. Agents used to reduce the amyloid plaque have shown less effect due to the presence of a blood-brain barrier. However, low-intensity focused ultrasound disrupts the blood-brain barrier and allows noninvasive, localized transport of imaging fluorophores and immunotherapeutic agents directly to the amyloid plaques [7, 48]. Tawfik et al. developed agomelatine-encapsulated invasions for the treatment of melatonergic antidepressants as an alternative drug delivery via the transdermal route to enhance its bioavailability, which confirmed significant improvement with sonophoresis technique as compared to oral delivery [106].

### 4.10. Sports Medicine

Sonophoresis is a universally approachable and extensively used electrotherapy in sports medicine. Ultrasound reduces the healing time of a fresh fracture up to 30–38%. This could be possible by using low-intensity pulsed ultrasound (LIPUS) < 0.1 w/cm^2^, a dose used in sports medicine, and is commonly used to cure injuries caused by tendons, ligaments, cartilage, and muscles [107, 108].

### 4.11. Sonothrombolysis

The ultrasound has a thrombolytic action, which is used in mechanical thrombolysis and used to improve enzyme-mediated thrombolysis. In the case of mechanical thrombolysis, a high-intensity ultrasound is used, which may cause some unnecessary side effects. Still, ultrasound in enzyme-mediated thrombolysis is much safer compared to diagnostic ultrasound. Currently employed, three different ultrasound-based therapeutic options are catheter-delivery tipped-ultrasound thrombolysis, catheter-delivered ultrasound transducer in thrombolysis, and transcutaneous noninvasive ultrasound thrombolysis [109]. Mechanical disintegration of the thrombus or activity of the administered thrombolytics is increased by using the physical effects of the ultrasound, like acoustic streaming, thermal effects, and shear stress. The formation of intracoronary thrombus characterizes acute coronary syndromes. However, coronary ultrasound thrombolysis is an effective and safe method to lyse the formed thrombus in saphenous vein grafts and acute myocardial infarction [110–114].

## 5. Challenges

### 5.1. Limitations of Sonophoresis

Even though sonophoresis is used to increase the permeability of the skin, it alone cannot provide enough efficiency for enhancing the permeation of large molecules. It also causes irritation, minor tingling, and burning sensation as skin is sensitive to varying temperatures. Sonophoresis is a time-consuming process, and the stratum corneum should be intact for effective drug transmission. A few drugs can be absorbed in a therapeutic dose, and sonophoresis alone is inefficient for enhancing macromolecule permeation [25, 115].

### 5.2. Challenges Faced by the Researchers

Sonophoresis has a vast scope for researchers. It is also shown that the distance between the skin and the transducer, cooling system, quality and type of coupling medium, processing of membranes, and the endpoint of evaluation of techniques affect the process of sonophoresis and its drug permeation rate. Usually, researchers have difficulty calibrating the amount of ultrasound energy emitted because ultrasound travels away from the source and expands after a particular critical distance. Technically, it is dependent on the radius of the transducer, ultrasonic wavelength, and effects, which are unified with constructive and destructive wave interference. There is a need to understand the ultrasound-tissue interaction by biophysical mechanism, which is not yet completely understood. This is due to the lack of understanding of certain phenomena that occur when skin is exposed to ultrasound; those simultaneously occurring phenomena are cavitation, convectional transport, mechanical effects, and thermal effects. If one of the dominant phenomena is understood, then it is easy to select an ultrasound parameter that enhances the favorable phenomenon, thereby increasing the efficacy of the system [107].

### 5.3. Safety Issues in Patients

After switching off the ultrasound in sonophoresis, the effect on skin barrier properties and underlying skin tissues describes its safety characteristics. According to many reports, the therapeutic application of ultrasound ranging from 1 to 3 MHz, 0–2 W/cm^2^ will not cause any permanent change in the skin barrier properties. Many reports related to safety measures of nonthermal and ultrasound bioeffects have been made in an experiment for adopting a policy related to safety guidelines issued by the World Federation for Ultrasound in Medicine and Biology. Many experiments have been conducted in clinical and laboratory studies to estimate the safety qualities of low-frequency ultrasound on exposure. No physical damage was observed on the skin on exposure to the ultrasound at a low intensity and frequency of 20 kHz. Some experiments reported that human skin exposed to low intensities of 2.5 W/cm^2^ showed no variation in vitro conditions, whereas intensities of 5.2 W/cm^2^ showed epidermal detachment and caused upper epidermal edema. However, using high intensities showed various side effects. Several parameters, such as intensity, frequency, application time, duty cycle, distance of the horn, and the type of tissue used, are vital for the safe application of low-frequency ultrasound in the clinical setup [107].

## 6. Conclusion and Future Trends

Sonophoresis is mainly based on the cavitation mechanism, which increases the permeability and penetration of drugs through the skin. Other mechanisms, such as thermal effects, convective transport, and mechanical effects, are studied, but the cavitation mechanism is highly focused and studied. The different applications in the pharmaceutical and biomedical fields, such as the transport of hydrophilic drugs, transport of macromolecules, gene delivery, vaccine delivery, nanocarrier-mediated delivery (micelles, liposomes, dendrimers), ocular drug delivery, nail delivery, sports medicine, sonothrombolysis, and also some of the synergistic effects of sonophoresis with permeation enhancers, are studied in brief. Sonophoresis is combined with other drug delivery techniques, which is used as an alternative to oral delivery and hypodermic injections for those drugs with less bioavailability, with more extensive or small molecules, which is required in small doses. Low-frequency sonophoresis-mediated drug delivery is effective in three significant areas: vaccination, for drugs with low bioavailability and gene delivery to the skin. The major challenges for the future of ultrasound-mediated drug delivery involve the quantity and kinetics of drugs that can be delivered transdermal, the price and size of the ultrasound equipment, and long-term safety studies, which are still required to be carried out and studied in detail. Long-term safety concerns concerning repeated treatment on the same site must be confirmed. The innovative delivery modalities involving two-frequency ultrasound and ultrasound contrast agents will assist in solving the permeabilization issue. At the same time, cymbal transducers will enable the low-cost manufacturing of lightweight, wearable devices.

## Figures and Tables

**Table 1:** The list of molecules showed the permeation using the sonophoresis technique.

Applications	Drugs	The outcome of the study	References
Drug delivery	Mannitol, dextran	A study reported that the passive permeability of dextran on ultrasound-treated skin is higher than that of mannitol	[31]
	Ophthalmic dosage forms	Sonophoresis enhances drug delivery by improving corneal permeability over a frequency of 20 kHz	[32]
	(i) Antibiotics		
	(ii) Steroids		
	(iii) *β*-blockers		
	Low-molecular wt. heparin (LMWH)	Skin permeability of LMWH was increased using 20 kHz frequency, which was more potential than the injections	[33]
	Salicylic acid	10 MHz frequency led to a 4-fold increase in penetration, whereas 16 MHz led to a 2.5-fold increase	[22]
	5-fluorouracil	Thermal effects of ultrasound accelerated 5-fluorouracil diffusion through the gel across the rat skin	[34]

Macromolecules	Linagliptin	Sonophoresis emerged as a promising physical enhancement technique for transdermal delivery of linagliptin, achieving a substantial increase in permeation (141.13 ± 34.22 *μ*g/sq. cm.), highlighting its potential as an effective strategy for enhancing drug delivery across the skin barrier	[35]
	Insulin, lidocaine	48 kHz frequency-enhanced permeation	[19, 36, 37]
	*γ*- interferon, erythropoietin	20 kHz frequency enhances the transfer	[38, 39]
	Basic fibroblast growth factor	This study demonstrates the efficacy of a transdermal delivery system using sonophoresis and carboxymethyl chitosan nanocarriers for administering basic fibroblast growth factor (bFGF) over a wide area of skin, offering a promising approach for enhanced delivery of biomacromolecules	[40]

Gene delivery	Naked plasmid encoding luciferase gene	A study has demonstrated that sonophoresis is a novel, nonviral, safe, and efficient gene transfer method into cultured vascular cells and blood vessels	[41]
	Cancer gene therapy to transduce herpes simplex thymidine kinase gene *in vitro*	A study reported that low-frequency ultrasound and nanobubbles reduced tumor size by a factor of four	[42]

Vaccine delivery	Cholera toxin	Applying ultrasound to the skin enhances the permeability of both adjuvant and vaccine	[36, 43]

*Nanocarriers*			
(i) Nanobubbles	5-fluorouracil and ascorbic acid	Ultrasound-responsive nanobubbles or bubble liposomes were used to deliver hydrophilic drugs to the brain via ultrasound. The pharmacokinetic study in mice and rats confirmed the intracerebral penetration	[26]

(ii) Micelles	DOX encapsulated in poly(ethylene glycol)-co-polycaprolactone (PEG-PCL) micelles	Ultrasound promotes DOX trafficking into cell nuclei, which is especially noticeable when nanoemulsions are present, which are converted into microbubbles by the action of ultrasound	[44]

(iii) Microneedles	Tetramethylpyrazine hydrochloride (TMPH)	The study comparing vibrating microneedles and low-frequency ultrasound for enhancing transdermal delivery of tetramethylpyrazine hydrochloride found that the vibrating microneedle system exhibited significantly higher efficacy, with a 41.92-fold increase in cumulative permeability compared to a 4.34-fold increase with low-frequency ultrasound	[45]

(iv) Liposomes	Doxil, Caelyx (doxorubicin in poly(ethylene glycol)-coated (sheath) liposomes)	Drug release through liposomes was increased by ultrasound and was found to be dependent on liposomal size and percent drug entrapment	[46]

(v) Dendrimers	Diclofenac	A study reported that the dendrimer-coupled sonophoresis increased diclofenac permeation by a factor of 16.5	[47]
	Low-molecular-weight dendrimers	The study demonstrated enhanced permeation of peptide dendrimers across human skin	[48]
